# Syrah: a pipeline to maximize spatial transcriptomics data output

**DOI:** 10.1093/g3journal/jkag107

**Published:** 2026-05-04

**Authors:** Carolyn Brewster, Frederick G Mann, Blair Benham-Pyle, Alejandro Sánchez Alvarado

**Affiliations:** Stowers Institute for Medical Research, 1000 E 50 St, Kansas City, MO 64110, United States; Stowers Institute for Medical Research, 1000 E 50 St, Kansas City, MO 64110, United States; Howard Hughes Medical Institute, 1000 E 50 St, Kansas City, MO 64110, United States; Stem Cells and Regenerative Medicine Center, Cell and Gene Therapy Center, Baylor College of Medicine, Baylor University, 1 Baylor Plaza, Houston, TX 77030, United States; Department of Molecular and Cellular Biology, Baylor College of Medicine, Baylor University, 1 Baylor Plaza, Houston, TX 77030, United States; Stowers Institute for Medical Research, 1000 E 50 St, Kansas City, MO 64110, United States; Howard Hughes Medical Institute, 1000 E 50 St, Kansas City, MO 64110, United States

**Keywords:** spatial transcriptomics, Slide-seqV2, Curio Seeker, bioinformatics resources

## Abstract

Spatial analysis of gene expression patterns has been a key technique for revealing the potential functions of genes. Traditionally, these analyses, conducted using *in situ* hybridizations and other labor-intensive protocols, were constrained to examining only a few candidate genes per sample. However, the advent of spatial transcriptomic techniques like Slide-seqV2 has transformed this field, enabling massively parallel exploration of gene expression patterns within their tissue contexts by pairing spatial locations with RNA sequencing. Despite its potential, Slide-seqV2 datasets often produce fewer usable reads than expected. We have identified that a significant source of errors in the technology stems from the chemical synthesis of barcodes used in Slide-seqV2. These errors are systematic, and in many cases, they can be bioinformatically identified and corrected. We have developed “Syrah,” an analysis pipeline that identifies and corrects barcode errors in Slide-SeqV2 and Curio seeker datasets. Syrah can dramatically enhance read numbers in Slide-seqV2 datasets, recovering up to 35% more reads, reassigning erroneous barcode matches, and removing improperly formed reads. Unlike other dataset improvement methods that rely on data-driven imputation, Syrah uses a biochemical model and the barcode sequence data and does not require additional datasets or intricate calculations. This innovative technique promises to transform the utility of Slide-seqV2 and Curio Seeker datasets by identifying usable reads that were discarded during previous analysis that required exact matching of barcode sequences.

## Graphical Abstract

**Graphical Abstract:**
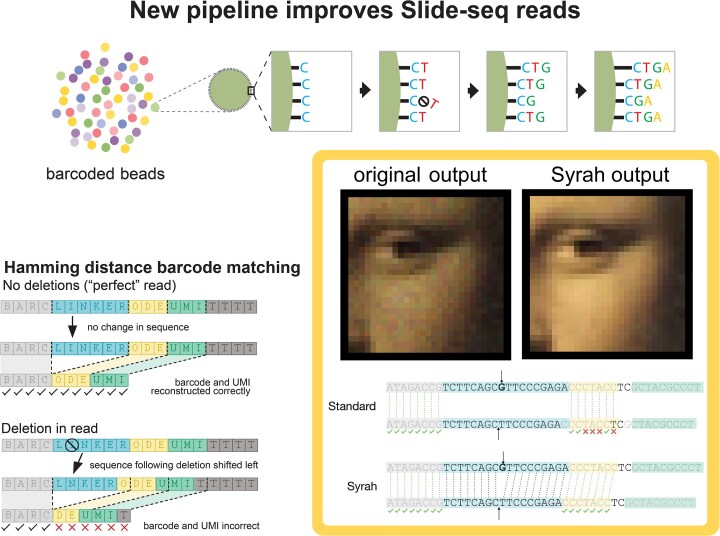

## Introduction

Understanding spatial patterns of gene expression in tissues is essential to understanding gene function. Whole-mount *in situ* hybridization has allowed us to visualize gene expression at the single-cell level in tissues *in vivo* (Pardue and Gall 1969; Tautz and Pfeifle 1989). However, this technique is limited to the characterization of a small number of target genes. Increasing the number of genes that can be evaluated for expression is what drives the development of spatial transcriptomics. Spatial transcriptomics is a family of technologies that provides novel biological insights about tissue composition and cell type interactions by localizing gene expression data to physical coordinates (Tian et al. 2023; You et al. 2024). Slide-seqV2 and Curio Seeker are spatial transcriptomic methods that use barcoded oligonucleotide-covered beads arranged in a monolayer on a glass slide to capture mRNAs from their immediate vicinity (Rodriques et al. 2019; Stickels et al. 2021).

**Fig. 1.**
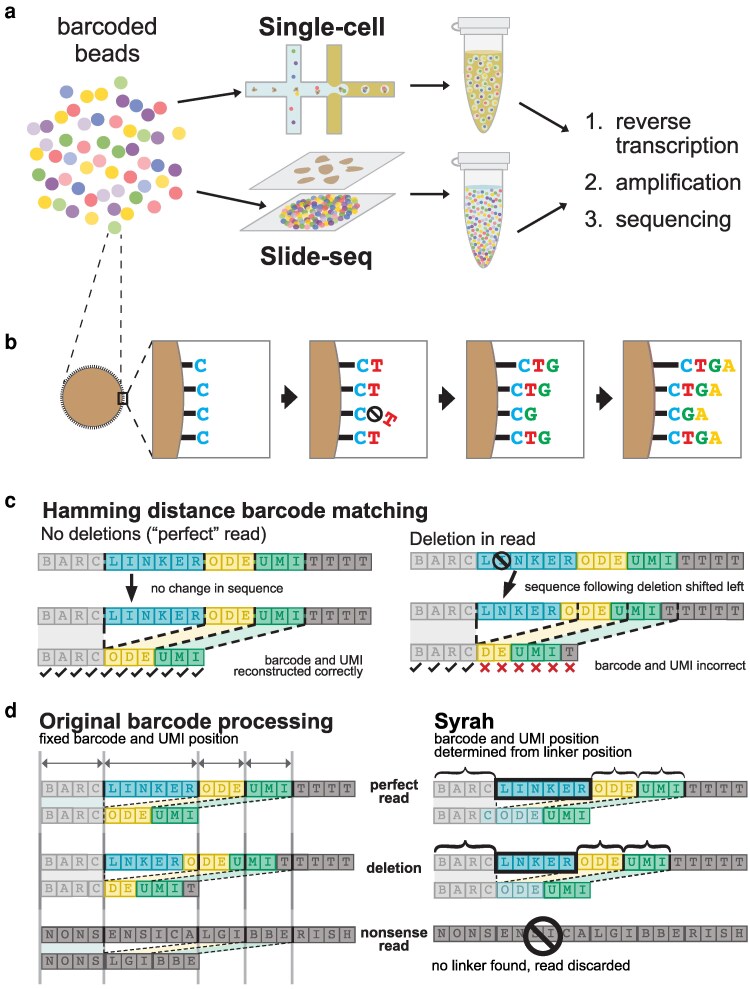
Errors occurring in bead-based transcriptomic data. a) Schematic of single-cell (top) and Slide-seqV2 (bottom) bead-based mRNA capture. b) Diagram of how incorporation failures during synthesis lead to sequence frameshifts. c) Illustration of how Hamming distance fails to correct for frameshifts. d) Diagram of how standard processing extracts barcodes from static positions (left), while Syrah uses the linker sequence to determine barcode location (right).

Slide-seqV2 and Curio Seeker generate spatially informed RNA-seq data by capturing mRNA with barcoded oligo dT beads very similar to the ones widely used for single-cell RNA-seq (Fig. 1a) (Macosko et al. 2015). This enables spatial analysis by relating each barcode to its *x*,*y* coordinates. The 10 μm diameter capture beads are arranged densely on a glass slide in a structure called a puck. The locations of each of the barcodes on the puck can be determined by performing SOLiD-like sequencing and microscopy (McKernan et al. 2009; Rodriques et al. 2019). Then, a tissue section is laid onto the puck, where beads capture mRNA and incorporate the spatial barcode when the mRNA is transcribed to cDNA (Fig. 1a, bottom). These data are extremely powerful and can identify gene expression patterns at near-cellular levels of detail, revealing biology that was previously inaccessible.

Popular single-cell technologies, such as Drop-seq, use mRNA capture beads where the oligonucleotide on the bead is synthesized via repeated phosphoramidite reactions that add a single nucleotide at a time (Fig. 1b) (Beaucage and Caruthers 1981; McBride and Caruthers 1983; Macosko et al. 2015). This synthesis strategy allows for combinatorial barcoding but also results in synthesis errors that cause frameshifts in the nucleotide sequence (Ellington and Pollard 2001 ; Zhou et al. 2004). This chemical reaction can only be driven to ∼99% efficiency. Slide-seqV2 and Curio-seeker use oligonucleotides produced in the same manner and subject to the same synthesis errors (Supplementary Fig. 1) (Fincher et al. 2018).

Once the barcoded beads are adhered to the “puck,” nondestructive SOLiD-like sequencing is used to produce a whitelist of bead barcodes and algorithmically assigns spatial coordinates to each bead it has detected. Ambiguities can occur during the microscopy-based sequencing that lead to virtually “duplicated” beads that share barcodes one substitution apart and spatial coordinates closer together than physically possible (less than 1 bead diameter, 10 µm).

Frameshifts during synthesis result in incorrect extraction of barcodes, even in cases where the “deleted” base was part of the linker sequence instead of the barcode itself. There are 3 downstream consequences of these barcode extraction errors. First, incorrect extraction of barcode sequences prevents a barcode match, leading to the loss of individual reads. Second, the resulting loss of read depth can cause the beads to fall below minimum UMI cutoffs, and entire beads can be excluded from the final data and appear as blank spots. Third, a barcode can be frameshifted to erroneously match a different barcode, causing a read to be incorrectly mapped onto an unrelated spatial location.

Additionally, standard processing pipelines use barcode matching algorithms that correct for substitutions, making them unsuitable to deal with sequence deletions. Syrah implements a barcode matching algorithm that flexibly handles deletions and/or substitutions to improve the rate and fidelity of barcode matching.

Here, we present Syrah, a computational tool that improves the quality and fidelity of Slide-seqV2 datasets by correcting for barcode error. Syrah is deployed as an R package for preprocessing error correction of bead-based spatial transcriptomics data using only the barcode map and the FASTQ file containing the barcode/UMI reads. The gains produced by Syrah can be extensive, increasing the total number of reads in some datasets by approximately 35%. The gains achieved through Syrah are distinct from, and complementary to, those obtained by deeper sequencing of Slide-seqV2 libraries, underscoring 2 crucial points: first, that Syrah can recover read data that would otherwise be discarded and excluded from downstream analysis, and second, that Syrah offers further improvement to datasets that have already undergone enhancement through other methodologies.

## Materials and methods

Slide-seqV2 facilitates the generation of unbiased spatial transcriptomic data via bead-based capture oligonucleotides. Each bead is designed to have a unique barcode synthesized in 2 parts with an invariant linker sequence between these 2 regions. Synthesis failures anywhere in this oligonucleotide will shift the position of the barcode within the read (Fig. 1c). This linker region allows the 2 parts of the barcode to be identified unambiguously, even if an oligonucleotide has suffered 1 or more failed incorporations during its synthesis (Fig. 1d). Existing approaches for handling imperfect barcode matches are not suitable for handling the frameshift errors caused by failed incorporations. Additionally, current methods use Hamming distance, which counts the number of positions at which 2 sequences differ (i.e. nucleotide substitutions) (Hamming 1950). Consequently, every position downstream of a single error can differ between the intended and actual sequence, leading to a very high Hamming distance score (Fig. 1c). It is possible to quantify differences between 2 sequences in more ways. For example, Levenshtein distance includes insertions and deletions in addition to substitutions (Levenshtein 1965). Such a metric would be more appropriate to address the failed nucleotide incorporations described above.

Syrah is a preprocessing pipeline implemented as an R library, with DBSCAN as the only required package. It is available on GitHub at https://github.com/0x644BE25/Syrah. Syrah is compatible with both Slide-seqV2 and Curio Seeker datasets but is not applicable to other spatial transcriptomic technologies (such as 10X Visium) where oligonucleotides of known sequence can be purified before conjugation to a substrate (Ståhl et al. 2016).

Code for the R library Syrah is available at https://github.com/0x644BE25/Syrah, while codes and additional data used for analysis presented in this manuscript are available at https://github.com/0x644BE25/Syrah_manuscript.

### Syrah implementation

**Fig. 2.**
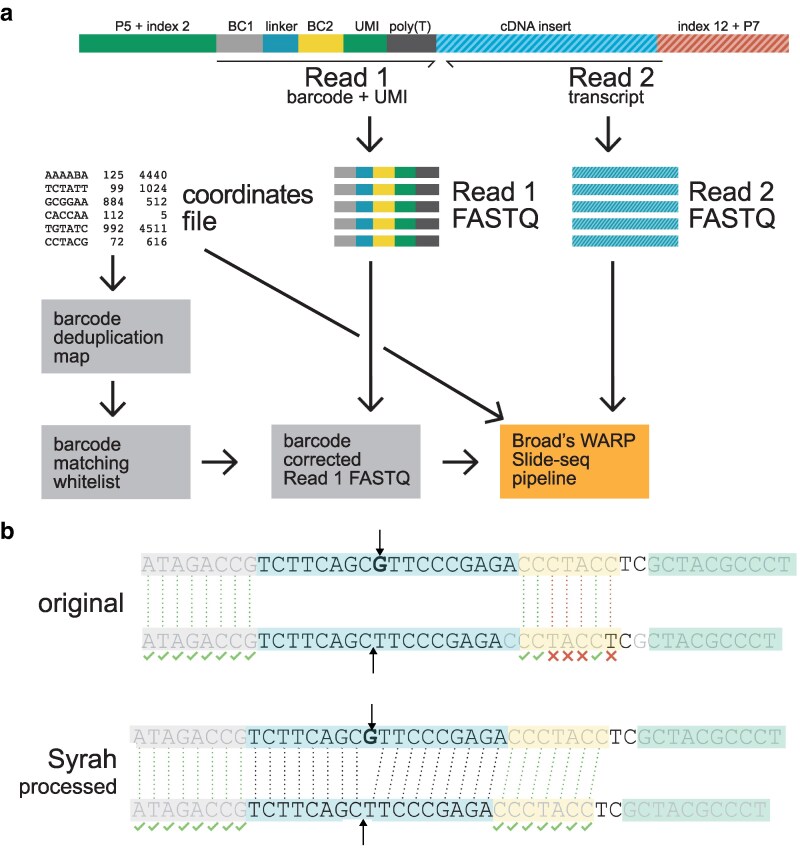
Syrah corrects for errors in Read 1 sequences. a) Schematic of Syrah's correction process. b) Illustration of effects of frameshifts on original (top) and Syrah-processed (bottom) barcode extraction.

Syrah requires as input 2 files: the bead coordinates file and the Read 1 FASTQ, which contains the portion of the read with barcodes and UMIs (Fig. 2a). Syrah does not alter—or require—the Read 2 FASTQ, which contains the sequence derived from the captured mRNA molecules. As such, it is agnostic to both the sample origin and the choice of reference transcriptome.

#### Generating a barcode matching whitelist

This step uses the bead coordinate file to identify virtually duplicated beads with barcodes 1 substitution apart and spatial coordinates closer than the physical bead radius should allow (<10 µm). For each group of duplicated beads, 1 bead is selected, and all other barcodes in that group are redirected to it. These data are saved as a bead deduplication map.

This step uses the bead deduplication map to generate a barcode matching whitelist that contains all matches that are 1 deletion or 1 substitution away from a single bead in the deduplication map. This whitelist is saved, and because it is derived from a model of the chemical synthesis process for the barcodes in a given library, it can be used to process all sequencing results for that library, such as in the case of a library being sequenced on multiple lanes.

#### Barcode extraction and matching

This step combines Read 1 data with the barcode whitelist to generate a new Read 1 FASTQ file that contains corrected barcode and UMI information. To correct for frameshifts, Syrah employs modified Levenshtein distance (allowing only substitutions and deletions) to find the position of the linker sequence and determine the barcode and UMI positions for extraction (Fig. 2b). Reads without an unambiguous linker sequence or with more than 1 deletion in the first half of the barcode are discarded. The extracted barcode is matched against the whitelist from the previous step, and the corrected barcodes are inserted into the corrected Read 1 FASTQ.

The corrected Read 1 FASTQ can be input into the WARP Slide-seqV2 pipeline along with the original coordinates file and Read 2 FASTQ. Syrah-corrected Read 1 FASTQ files should be compatible with any pipeline that analyzes Slide-seqV2 or Curio Seeker data.

### Planarian dataset generation

The planarian data were generated from planarians embedded in OCT histology media, fresh frozen, and sectioned directly onto a Slide-seqV2 puck, following the protocol of a recent study (Mann et al. 2025). After library preparation, the planarian data in this manuscript were sequenced twice for the analysis of sequencing depth on data quality.

### Additional species datasets

We identified 3 published datasets with full Read 1 sequence data (Supplementary Table 1): a chick Slide-seqV2 dataset, a human Curio Seeker dataset, and the mouse spleen test data provided with the Curio Seeker pipeline (Kasemeier-Kulesa et al. 2023; Feng et al. 2025).

### Data processing

All datasets were run through the Syrah pipeline, and both original and Syrah-processed versions were processed with the Broad WARP Slide-seqV2 pipeline v3.1.2 with default parameters. The planarian dataset was aligned to the schMedS3 genome (Grohme et al. 2018). The chick dataset was aligned to the *Gallus gallus* genome assembly bGalGal1.mat.broiler.GRCg7b retrieved from Ensembl release 115 and available at https://e115.ensembl.org/Gallus_gallus (Harrison et al. 2024). The human dataset was aligned to the human genome assembly GRCh38.p10 available at https://www.gencodegenes.org/human/release_27.html (Schneider et al. 2017). The Curio-seeker mouse dataset was aligned to the GRCm38.p6 mouse genome assembly available at https://www.ncbi.nlm.nih.gov/datasets/genome/GCF_000001635.26/ with Gencode M23 annotations (Church et al. 2009, 2011). Script at https://github.com/0x644BE25/Syrah_manuscript/blob/main/WARP_non-default_parameters.json.

### Clustering

Sparse format gene expression matrices from the WARP pipeline were processed with the R library Seurat, filtered to beads with at least 50 UMIs, and normalized with SCTransform. Principal component analysis was performed (planarian 15 PCs, mouse 5 PCs, chick 15PCs, human 10 PCs), followed by FindNeighbors, FindClusters, and RunUMAP with default settings. Random seed was set to 61,687 for replicability. Script at https://github.com/0x644BE25/Syrah_manuscript/blob/main/multi-species_comparison.R.

### Differential expression analysis and cell type prediction

For cluster-level marker gene/differential expression analysis of planarian data, we used Seurat's FindAllMarkers to find differentially expressed genes for each cluster and filtered the results with a multiple-comparison-corrected *P*-value less than 0.05. For cluster-agnostic differential expression analysis, planarian data were partitioned into beads positive or negative for a canonical cell type marker (*piwi-1* for stem cells, *mat2a* for intestinal cells), and Seurat's FindMarkers was used to find significantly (multiple-comparison-corrected *P* < 0.05) differentially expressed genes with higher expression on the beads of interest. These genes were then compared to lists of known planarian cell type markers from a single-cell RNA-sequencing atlas (Benham-Pyle et al. 2021). Seurat's LabelTransfer functionality was used to predict cell type based on the unirradiated planarian single-cell RNA-seq data from Benham-Pyle et al. 2021. Script at https://github.com/0x644BE25/Syrah_manuscript/blob/main/additional_sequencing_DE_analysis_and_cell_type_predictions.R.

### Controlled deletion rate experiment

One million unique “perfect” reads were extracted from the planarian dataset. These are reads where the Read 1 structure is exactly as designed, with no deletions in the barcode or linker, and where the barcode matches perfectly to one on the puck. Deletions were randomly introduced into Read 1 with various probabilities using R's “sample()” function, shifting the remaining sequence left, and backfilling with poly(T) sequence. An additional dataset was created where the Read 1 sequence is entirely randomized for each read. This was repeated independently on all 1 million reads for each deletion rate (and random) with *n* = 5. Both original and Syrah-processed versions of the data were analyzed with the WARP pipeline identically to other planarian data. Script at https://github.com/0x644BE25/Syrah_manuscript/blob/main/deletion_rate_in_silico_experiment.R.

### Synthetic datasets from images

Synthetic Slide-seqV2 datasets were produced from images as follows. Images were scaled to 300 × 300 pixels, adding blank space as needed. Each pixel functioned as a stand-in for a bead, and hence, each pixel location was assigned a randomly generated barcode. Three single-mapping 100-nucleotide sequences from the Gencode M23 mouse genome were chosen to represent red, green, and blue (Supplementary Table 2). RGB color information on a scale of 0 to 255 was extracted for each pixel, and the number of reads for each of the transcripts was generated with that pixel's barcode. Errors were added randomly to Read 1 at 1% rate, and the missing sequence was backfilled at the 3′ end with poly(T). The generated FASTQs were processed with Syrah, and both the original and Syrah-processed data were analyzed using the WARP Slide-seqV2 pipeline on Terra, and aligning to the standard WARP mouse reference GRCm38 (Church et al. 2009, 2011). Both original and Syrah-processed versions of the resulting data were filtered to the same minimum UMIs per bead (determined individually for each image's data). Filtered data were processed with Seurat and used the assigned red, green, and blue gene expression data to find neighbors, run clustering, and generate a UMAP with default settings. Individual beads’ color data were reconstructed from expression data using the raw number of UMIs corresponding to red, green, and blue using an 8-bit RGB color scale. Script and input images at https://github.com/0x644BE25/Syrah_manuscript/blob/main/image-based_in_silico_experiment.R.

### Benchmarking

Benchmarking was performed using the Curio Seeker test data and a 1 million read subsample of the planarian Slide-seqV2 data. All tests used a single CPU core. The 4 computational environments tested were (1) an Apple M2 MacBook Air running MacOS Sequoia 15.5 (“mac”), (2) a custom PC running Windows 11, (3) a Lenovo ThinkPad T480s running Ubuntu 24.04.3 LTS, and (4) the Stowers Institute High Performance Computing cluster running Rocky Linux 9.6. The syrah() function is a wrapper for 3 functions: make_bead_dedup_map(), make_barcode_whitelist(), and correct_barcodes(). For each of 5 replicates, these functions were run sequentially with runtime and peak RAM usage measured. Script at https://github.com/0x644BE25/Syrah_manuscript/blob/main/benchmarking.R.

### Poisson rate testing

To compare the number of UMIs, beads, or genes between different versions of a dataset (i.e. Original vs Syrah-processed or doubly vs singly sequenced), we used a 2-sample Poisson rate test to compare the rate of “successful” inclusion in the final analyzed data, where the number of “successes” was the number of UMIs, beads, or genes present in the final analyzed data. We used R's poisson_test() function to perform a 2-sided Exact Poisson test with a confidence level of 95%.

## Results

**Fig. 3.**
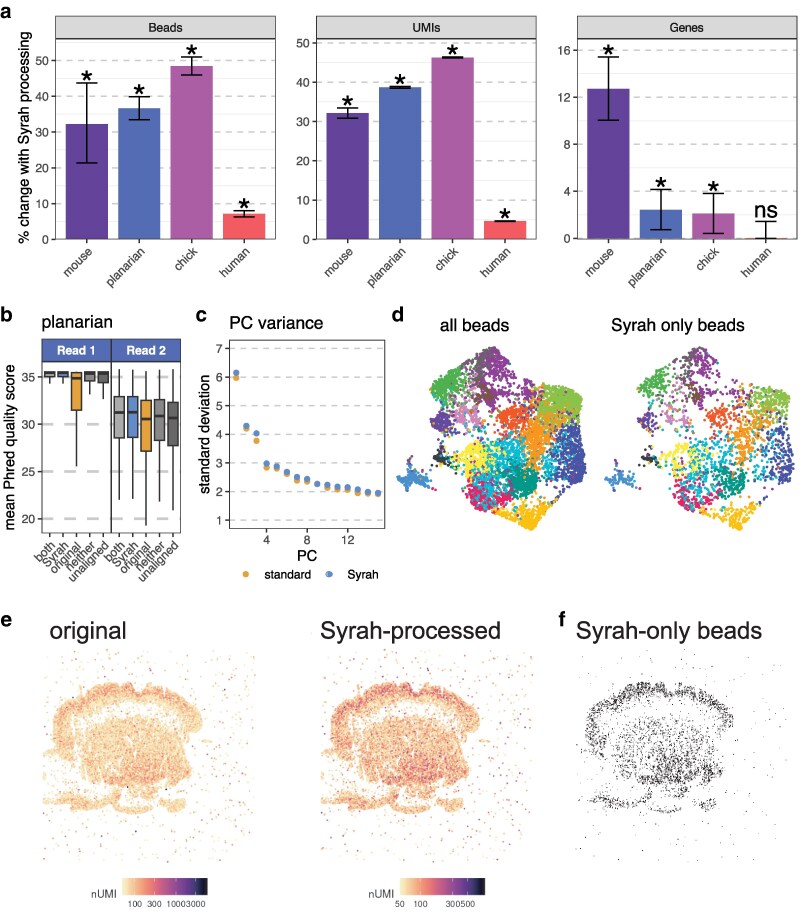
Syrah improves data across a variety of organisms and datasets. a) Percent change in total beads (left), UMIs (middle), and genes (right) for original vs Syrah-processed data across a variety of Slide-seqV2 and Curio Seeker datasets (Supplementary Table 1) with minimum 50 UMIs/bead cutoff. Bars indicate 95% confidence interval of Poisson rate test; asterisks denote *P*-value < 0.05. b) Boxplot of PHRED quality scores for planarian reads categorized by location in final data. Horizontal bar indicates median, boxes stretch from first to third quartile, and whiskers indicate smallest/largest nonoutlier values. Outliers are included in calculations but omitted from plot for visual clarity. c) Plot of standard deviation of principal components for original vs Syrah-processed planarian data. d) UMAPs of planarian Syrah-processed data colored by clusters generated using default parameters. Left UMAP shows all beads, right UMAP shows beads specific to Syrah-processed data. e) Slide embedding of the human dataset with beads colored by number of UMIs for original (left) and Syrah-processed (middle) data. f) Slide embedding of human dataset showing beads present only in the Syrah-processed dataset.

We tested Syrah on a variety of Slide-seqV2 and Curio Seeker datasets across a range of organisms (Supplementary Table 1). For each dataset, both original and Syrah-processed versions of the data were analyzed using the official WARP Slide-seqV2 pipeline available on Terra with default settings other than count_exons = false. Examples of the effects on individual reads are illustrated in Supplementary Fig. 2. Poisson rate tests were used to compare the likelihoods of UMIs, beads (≥10 UMIs), or genes appearing in the Original vs Syrah-processed version of the data. For nearly all cases, Syrah provided a significant increase in beads, UMIs, and genes recovered, although the increase in genes in the human dataset did not reach statistical significance (Fig. 3a, Supplementary Fig. 3a).

To evaluate the quality of the rescued reads included by Syrah, we compared the PHRED sequencing scores for reads rescued by Syrah, reads removed by Syrah, and reads shared between both the original and Syrah-processed datasets. Reads that were exclusive to the planarian Syrah-processed dataset showed a small increase in mean quality vs shared reads (Read 1: +0.0146, *P* < 0.00004;Read 2: +0.03049, *P* < 0.0075),, while reads exclusive to the original data (removed in the Syrah-processed version) had lower quality scores (Read 1: −1.76336, *P* < 10^−160^; Read 2: −0.86273, *P* < 10^−26^) (Fig. 3b). Similar effects were observed for the datasets from other organisms (Supplementary Fig. 3b, e, and h). For further analysis, the data were clustered and visualized using Seurat. First, we compared the principal components for the Original vs Syrah-processed versions of each dataset and found that the variance explained by each PC is comparable (chick, human) or better (planarian, mouse) for the Original vs Syrah-processed version of the data (Fig. 3c, Supplementary Fig. 3c, f, and i). We also reviewed the clustering results, finding that the new beads added by Syrah appear intermixed among the rest of the beads, indicating they likely contain meaningful additional data (Fig. 3d, Supplementary Fig. 3d, g, and j). Visualizing the original and Syrah-processed human datasets in their spatial embeddings shows that reads and beads gained in the Syrah-processed version correspond to the location of tissue in the original embedded section (Fig. 3e and f). Together, these results indicate Syrah can improve spatial transcriptomic datasets by rescuing high quality reads, beads, and adding genes that enhance the biological signatures captured by the experiment.

### Runtime statistics

**Fig. 4.**
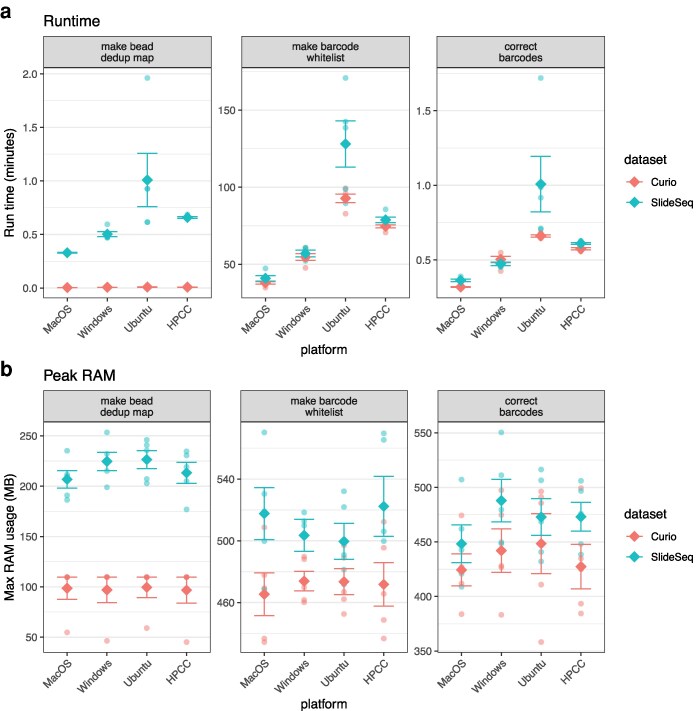
Runtime and RAM benchmarking. Benchmarking of Syrah's a) run time and b) peak RAM usage using Curio Seeker and Slide-seqV2 test data each with 1 million reads. Points indicate individual results, diamonds represent means, and error bars show mean standard error.

All benchmarking was performed using a single CPU core. The first step of Syrah is finding the duplicated beads, which generally takes less than 5 min. The second step is revising the barcode whitelist. This usually takes less than an hour (using a single core) for Slide-seqV2 datasets (Fig. 4). Because Curio tiles can be far larger, whitelist generation may take roughly 24 h for the largest tiles. For any given puck or tile, the whitelist only needs to be generated once and can then be used for any number of FASTQs from that library. The third step, barcode correction, scales linearly with the number of reads and is thus dependent on the depth of sequencing (Fig. 4). I/O processes predominate at smaller dataset sizes, but for larger dataset sizes, Syrah's runtime scales linearly (Supplementary Fig. 4a), and it generally processes between 1 and 3 million reads per minute (Supplementary Fig. 4b).

### Comparison between Syrah and increasing sequencing depth

**Fig. 5.**
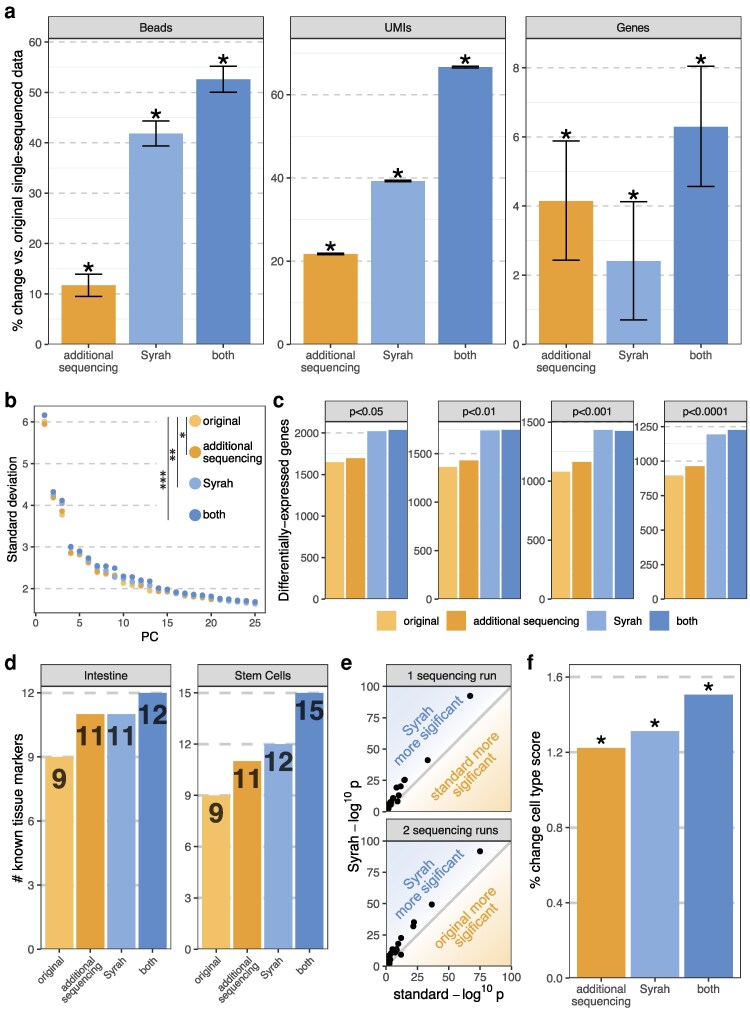
Syrah provides improvements greater than and orthogonal to additional sequencing. a) Bar plots showing the percent increase in beads (left), UMIs (middle), and genes (right) provided by additional sequencing, Syrah processing, or the combination of additional sequencing and Syrah processing on the planarian dataset. Bars indicate 95% confidence interval of Poisson rate test of Syrah-corrected vs original data from a single sequencing run; asterisks denote *P*-value < 0.05. b) Principal components calculated and used by Seurat for clustering the 4 versions of the planarian data. Asterisks indicate *P* < 0.05 for paired 2-sample t-tests between versions of the data. All pairwise comparisons are significant. c) Total positive marker genes identified by Seurat's FindMarkers() function for each version of the planarian data. Statistical cutoffs for marker significance are indicated at the top of each plot. d) Bar plot of cell-type-specific planarian genes found to be positively differentially expressed on beads positive for a canonical intestine (left) or stem cell (right) marker. e) Comparison of statistical significance in the form of −log10 *P*-value for known cell type markers present in both original and Syrah-processed planarian data for singly sequenced (top) and doubly sequenced (bottom) datasets. f) Change in max cell type score vs original singly sequenced planarian data. Asterisks indicate *P* < 0.05 from a 2-sample *t*-test.

To compare the efficacy of Syrah vs additional library sequencing, we performed a second round of sequencing on our planarian library samples and also processed this doubly sequenced data with Syrah to generate 4 datasets: “original” (singly sequenced non-Syrah-processed), “additional sequencing” (double-sequenced, not Syrah-processed), “Syrah” (singly sequenced and Syrah-processed), and “both” (double-sequenced and Syrah-processed). The 2 rounds of sequencing identified 35 million and 37 million UMIs. A Poisson rate test was used to compare the likelihoods of beads, UMIs, and genes being included in each dataset, and we found that Syrah significantly increases these by 41%, 39%, and 3%, respectively (Fig. 5a). While additional sequencing provided gains in beads, UMIs, and genes, Syrah's gains in beads and UMIs were significantly larger. We also evaluated whether Syrah's benefits outperformed those of additional sequencing across a range of minimum cutoffs for UMIs per bead and found that this trend is consistent (Supplementary Fig. 5). Notably, Syrah extends gains for the dataset with additional sequencing, indicating that Syrah's improvements are orthogonal to additional sequencing and that it can recover reads that sequencing alone cannot.

### Syrah improves downstream analysis

To determine how Syrah impacts downstream analysis, we analyzed the statistical strength of differentially expressed genes that could be identified from the data. We performed this analysis for both original and Syrah-processed versions of the singly and doubly sequenced planarian data. The datasets were identically processed (see Methods) and clustered with default parameters. The standard deviations of the principal components used for the clustering were significantly larger for the Syrah-processed than the original version of the single sequencing run data, and this was also the case for the doubly sequenced data (Fig. 5b). We then used Seurat's FindAllMarkers function to find differentially expressed genes for all clusters across a series of statistical thresholds. The total number of differentially expressed genes identified at the *P* < 0.05 cutoff across all clusters was 1,646 for the original singly sequenced data and 2020 for Syrah-processed singly sequenced data (Fig. 5c). This trend was observed at all significance thresholds tested, with Syrah-processed data yielding approximately 20% more marker genes in each case. An additional round of sequencing only increased the number of marker genes to 1,697 at the *P* < 0.05 cutoff for original data and 2035 for Syrah-processed data. This trend was observed across all significance thresholds.

To test the effects of Syrah on cell type identification, we took 2 approaches. First, we investigated planarian data for differential gene expression between beads-positive and -negative canonical cell type markers for 2 of the most well-characterized planarian cell types: *piwi-1^+^* stem cells and *mat2a^+^* intestinal cells. We compared our lists of differentially expressed genes to lists of established marker genes for that cell type (Supplementary Table 3). We detected 11 intestinal markers in the Syrah-processed planarian data as compared to 9 in the original planarian data, and 12 stem cell markers for Syrah-processed data compared to 9 with the original data (Fig. 5d). As with other results, Syrah-processed data were equal to or outperformed additional sequencing alone and provided additional benefits when combined with sequencing. For marker genes that were shared between the original and Syrah-processed versions, the statistical significance was nearly always higher for the Syrah-processed versions version (Fig. 5e).

Our second approach to cell type identification was to use Seurat's LabelTransfer feature to determine the most likely cell type(s) contributing mRNA to each bead (see Methods). The average maximum cell type scores for each bead—representing statistical confidence in a cell type signature—were significantly higher for both additionally sequenced original data (+1.22%) and Syrah-processed singly sequenced data (+1.31%), but Syrah processing provided larger gains, and Syrah processing also improved the doubly sequenced data (+1.51%) (Fig. 5f). Together, these data demonstrate how the use of Syrah can strengthen and facilitate the analysis of data and improve the signal of underlying biology.

### Syrah successfully corrects for Read 1 deletion errors

**Fig. 6.**
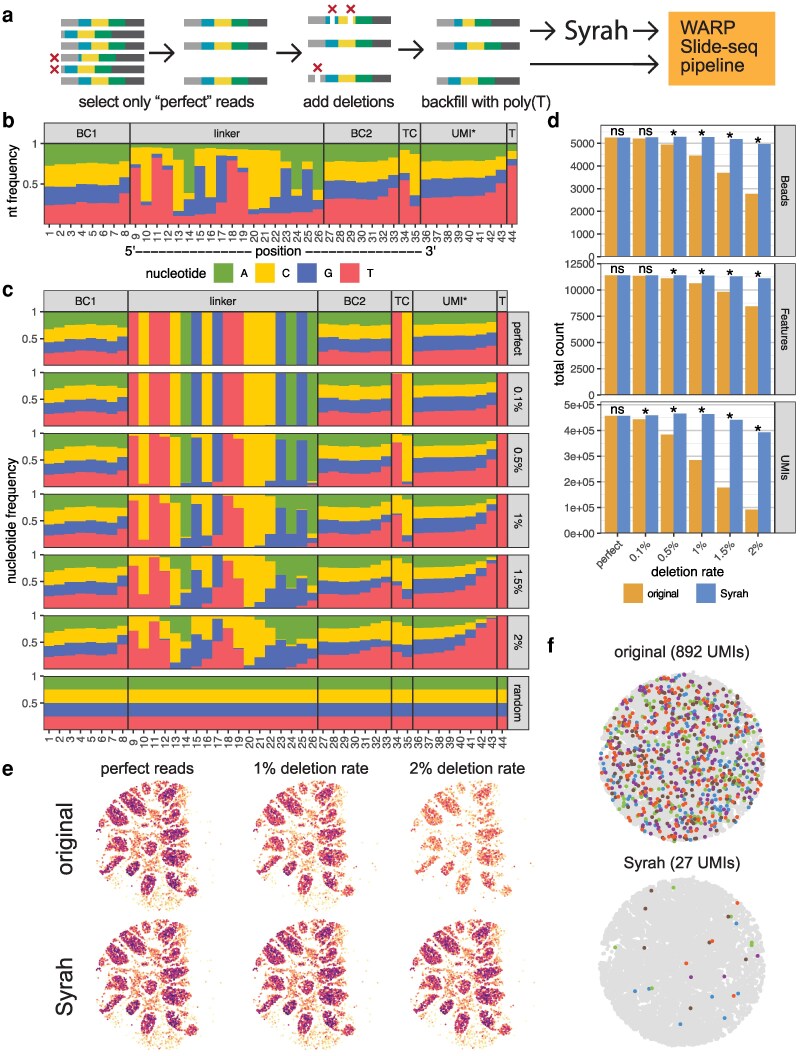
Syrah improves data across a range of Read 1 deletion rates. a) Schematic of *in silico* experiment that introduces deletions into perfectly structured Read 1 sequences. b) Nucleotide frequency by position for planarian data from which perfect reads were extracted. c) Nucleotide frequency across Read 1 position for datasets with various deletion rates. Bottom row shows data with completely random Read 1 sequences. d) Bar plots of total beads (top), UMIs (middle), and genes (bottom) for original and Syrah-processed versions of data across various deletion rates (minimum 10 UMIs per bead). Asterisks denote Poisson rate test *P*-value < 0.05. E) Slide embeddings of original (top) and Syrah-processed (bottom) data with either perfect reads (left), 1% deletion rate (middle), or 2% deletion rate (right) colored by UMIs per bead. e) Slide embeddings of original (top) and Syrah-processed (bottom) data with beads colored by the number of UMIs. f) Slide embeddings of planarian data with beads from perfect read data in gray. Colored dots indicate beads where completely randomized reads were assigned, with color denoting replicate.

To test the effectiveness and fidelity of barcode correction across a variety of conditions, we performed an *in silico* experiment where deletions were introduced to the Read 1 sequence in a controlled manner (Fig. 6a). This allows us to evaluate Syrah's benefits in a context where the ground truth is known; these reads have perfect structure and barcodes that match perfectly to a single bead on the puck. We can then test the effects of various artificially introduced deletion rates on data processed with and without Syrah processing and assess Syrah's ability to overcome these introduced errors. To create this dataset, we extracted 1 million unique “perfect” reads from the planarian dataset. These are reads where the Read 1 structure is exactly as designed, with no deletions in the barcode or linker, and where the barcode matches perfectly to 1 on the puck. We then created different experimental conditions by introducing deletions into Read 1 with various probabilities ranging from 0.1% to 2% and backfilling with poly(T) sequence. Each experimental condition was replicated 5 times, with deletions introduced each time independently. As a negative control, a dataset was created where the Read 1 sequence is entirely randomized for each read. Both original and Syrah-processed versions of the data were analyzed using the WARP Slide-seqV2 pipeline.

We first sought to investigate whether the *in silico* data recapitulate the oligonucleotide sequences we observe in real unfiltered data. Read 1 nucleotide frequencies plotted by position for original planarian data show the same pattern of frameshifting seen in the data with introduced deletions and suggest that the original dataset had a deletion rate of approximately 1% (Fig. 6b and c). Comparison of beads, UMIs, and genes across the 5 replicates of each deletion rates shows that Syrah's benefits in all 3 categories are directly proportional to the rate of deletions in Read 1 and that Syrah is capable of salvaging most reads even at high deletion rates (Fig. 6d and e, Supplementary Fig. 6). For a 0.5% deletion rate, Syrah increased beads by 6% UMIs by 21%, and genes by 2%. These improvements increased to 18%, 62%, and 6%, respectively, and a 2% deletion rate yielded 82% more beads, 327% more UMIs, and 32% more genes (Fig. 6d). Syrah also reduces noise by preventing spurious barcode matches from poorly formed reads: while the original version had 188 of the randomized reads assigned to beads, the Syrah-processed data only had 5 (Fig. 6f, bottom).

### Syrah improves data fidelity and reduces noise

**Fig. 7.**
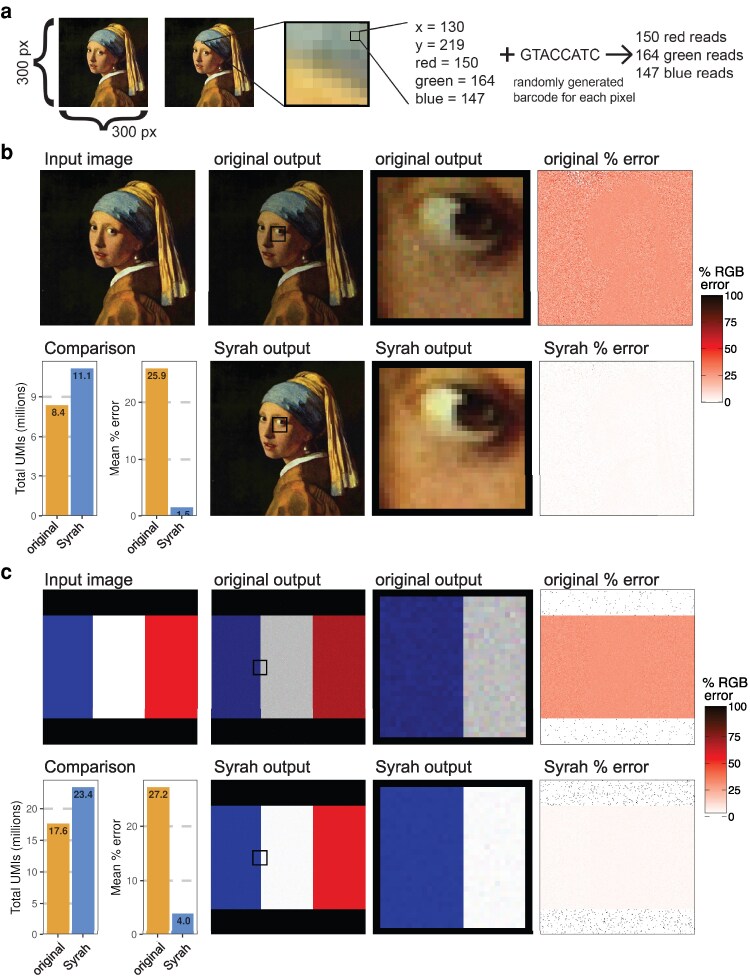
Syrah improves quantity and fidelity of synthetic image-based data. a) Schematic of image-based spatial dataset generation. b,c) Two image-based de novo spatial datasets. Top left is starting image data. Bottom left shows total UMIs and mean percent RGB color error for both original and Syrah-processed versions. Center-left column shows slide embedding for original (top) and Syrah-processed (bottom) data colored by individual bead RGB values. Center-right column is detail inset of slide embedding. Right columns show slide embedding of original (top) and Syrah-processed data colored by % RGB error for each bead.

To test the downstream effects of Syrah's error correction, we constructed synthetic datasets where the ground truth could be known *a priori*. To facilitate a visual representation of Syrah's performance, we generated these datasets from 4 familiar images of varying complexity: Johannes Vermeer's painting *Girl with a Pearl Earring* (Fig. 7b, top left), the French national flag (Fig. 7c, top left), clip-art of a bowl of fruit (Supplementary Fig. 7a, top left), and Leonardo Da Vinci's *Mona Lisa* (Supplementary Fig. 7b, top left). In this visual metaphor, each pixel represents a spatial bead, and the red/blue/green intensity values represent expression data for 3 genes (Fig. 7a). Synthetic barcodes were assigned to each pixel, with deletions introduced at a 1% frequency. The 4 resulting datasets were processed with Syrah, and both the Syrah-processed and original versions of the data were run through the WARP pipeline. In all 4 cases, Syrah improves the quality of the datasets by increasing the total number of UMIs (image brightness) by 32.6%–33.1% and by reducing color distortion (as compared to the original image) by 83.9%–94.2% (Fig. 7b and c; Supplementary Fig. 7).

## Discussion

Syrah is a data preprocessing algorithm that is easy to run and does not require any additional data (such as single-cell RNA-seq) to function. While there exist many methods for augmenting Slide-seqV2 and Curio Seeker data by imputation, these use probability to predict the transcriptome of cells from the sample (35 methods are listed in Supplementary Table 4). These methods operate on the “Read 2” data containing the transcript sequence, functioning to deconvolute mixed profiles from 2 or more cells. In contrast, only Syrah is a preprocessing tool that operates on the “Read 1” barcode+UMI sequence, enabling the recovery of additional sequencing reads that were lost by standard preprocessing steps. Both ways of improving such datasets need not be mutually exclusive: it seems quite likely that Syrah could make other methods perform better by increasing the amount of empirical data on which to work. We showed that the gains produced by Syrah exceeded those from additional sequencing, and preprocessing with Syrah plus additional sequencing provided synergistic benefit. We anticipate that Syrah will allow researchers to rescue languishing or discarded Slide-seqV2 and Curio Seeker datasets.

Syrah improves Slide-seqV2 datasets by correcting for errors introduced by bead forking and failed incorporations during nucleotide synthesis. These errors are unavoidable consequences of the fact that the best available method for nucleotide synthesis, the phosphoramidite reaction, cannot be driven to 100% efficiency (Beaucage and Caruthers 1981; McBride and Caruthers 1983; Ellington and Pollard 2001; Carr et al. 2004; Zhou et al. 2004; Binkowski et al. 2005). For conventional base-by-base synthesis, there is simply not a better option to synthesize oligonucleotides onto solid supports, including the beads used by Slide-seqV2. One emerging possibility might be for future technologies to rely on polymerase-based nucleic acid synthesis. High-fidelity DNA polymerases can have error rates as low as 10^−7^ per base, offering the possibility of nearly error-free copying of oligo templates onto a solid support (Potapov and Ong 2017). However, such a method requires a highly pure template to start, carefully engineered solid supports to allow PCR amplification of the template, and potentially expensive equipment to perform the reaction (such as running it on a sequencing flowcell or equivalent). Direct synthesis onto solid supports will likely remain a key chemistry method for some time, requiring correction methods like those implemented in Syrah.

The complications caused by the limitations of the phosphoramidite sequencing reaction are not unique to Slide-seqV2 (Septak 1996; Binkowski et al. 2005; Kosuri and Church 2014). Any technology that depends on oligonucleotides synthesized in this way has the potential for its downstream data to be improved by correcting for these incorporation errors. A few examples include the commercialized version of Slide-seqV2, Curio's “Seeker,” the widely used Drop-seq, and HIVE-seq from Honeycomb Biotechnologies (Macosko et al. 2015; Gierahn et al. 2017; Stickels et al. 2021). Differences in oligonucleotide structure and ground-truth barcode determination will require adaptation of Syrah's correction methods to specific technologies.

While the overall length of the bead-bound Slide-seqV2 capture oligos made them particularly susceptible to suffering “deletions” from failed incorporation of nucleotides at each position, their inclusion of an invariant region of defined sequence substantially supported our efforts to correct for the problem. If the linker had been absent or comprised of random nucleotides, we would not have been able to use alignment relative to the linker to select the proper “reading frame” from which to extract the barcode. Technologies that require direct synthesis of oligonucleotides onto solid supports are constrained by the limitations of the available synthesis chemistries (LeProust et al. 2010; Roy and Caruthers 2013). Those that rely on base-by-base synthesis through the phosphoramidite (or similar) methods will be subject to the same deletion effects we have described here (Septak 1996; LeProust et al. 2010; Kosuri and Church 2014). We suggest that the inclusion of an invariant sequence in the oligo would enable future correction of data produced from these technologies and should be considered a best practice for the design of such tools.

### Limitations of Syrah

Syrah can improve the depth and quality of many spatial transcriptomics datasets, but it cannot overcome certain systematic errors during the handling and preparation of the tissue samples. If the extent of failed incorporations during synthesis is so high that many reads have linker sequences that Syrah cannot recognize (i.e. 6 or more deletions in the 18-base linker), these reads will be unsalvageable. Syrah also cannot rescue reads where nucleases have degraded the transcript beyond the point where it can be mapped. Heavily blurred data produced by dragging the tissue sample across the puck would be of limited value for spatial analysis, although Syrah would be able to deepen it by rescuing discarded reads.

## Conclusion

Syrah improves analysis of spatial transcriptomics data by rescuing lost reads and beads. It is fast and simple to run, operates only on the bead map and barcode/UMI read, and does not require any additional datasets to perform its functions. Syrah maximizes spatial transcriptomics data output by increasing read depth and preventing or correcting erroneous mapping of reads, and this improves biological signal strength and fidelity in downstream analysis.

## Supplementary Material

jkag107_Supplementary_Data

## Data Availability

Planarian data are available at GEO accession GSE289299. Chick data are SRR24325152 from GEO accession GSM7234197. Human data are SRR31095896 from GEO accession GSM8591094. The Curio test dataset is the test data originally provided with the Curio Seeker pipeline and is now mirrored on the Syrah GitHub at https://github.com/0x644BE25/Syrah/raw/refs/heads/main/test_data/example_input_mouse_spleen_1M.tar.gz for convenience. Additional information about datasets is available in Supplementary Table 1. Statistical *P*-value data are available in Supplementary Table 5. Syrah pipeline: The Syrah R library and instructions for installation, use, and troubleshooting are available on GitHub. Project name: Syrah. Project home page: https://github.com/0x644BE25/Syrah. Operating system(s): Platform independent. Programming languages: R. License: GNU GPL. Any restrictions to use by nonacademics: none. Syrah manuscript: The authors affirm that all data necessary for confirming the conclusions of the article are present within the article, figures, and tables. The code used to perform the analyses can be freely accessed below: Project name: Syrah manuscript. Project home page: https://github.com/0x644BE25/Syrah_manuscript. Operating system(s): Platform independent. Programming languages: Bash, R. License: GNU GPL. Any restrictions to use by nonacademics: none. Supplemental material available at G3 online.
