# Supplementary material for: Syrah: a pipeline to maximize spatial transcriptomics data output
Source: G3 (Bethesda). 2026 May 4;16(7):jkag107. doi: 10.1093/g3journal/jkag107 (PMC13334189; doi:10.1093/g3journal/jkag107)
Supplement: jkag107_Supplementary_Data [file jkag107_supplementary_data.zip › Figure_S7_G3-2025-406488R1.pdf]

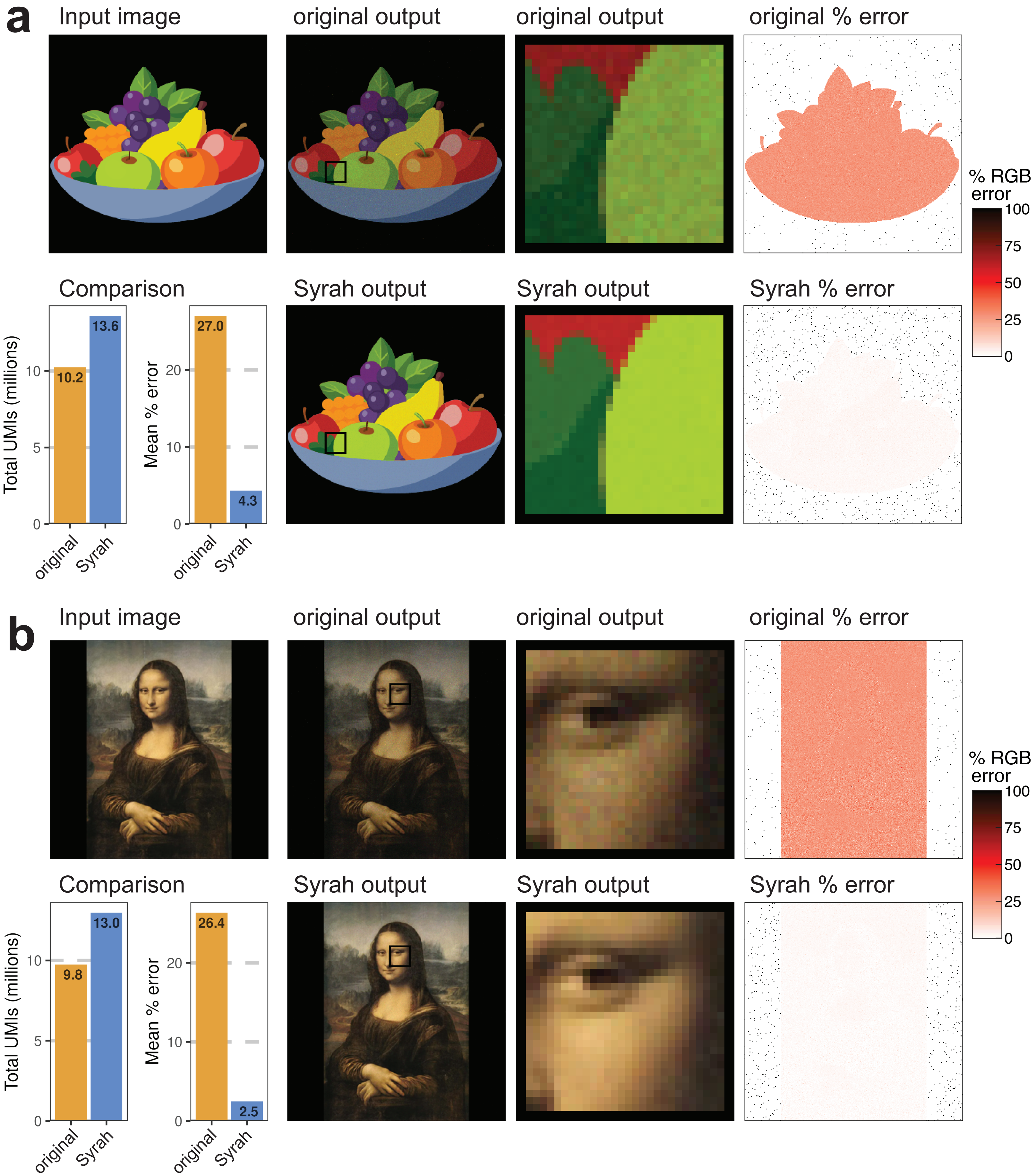
a
Input image
original output
original output
original % error
% RGB error
100
75
50
25
0
Comparison
Total UMIs (millions)
10.2
13.6
original
Syrah
Mean % error
27.0
4.3
original
Syrah
Syrah output
Syrah output
Syrah % error
b
Input image
original output
original output
original % error
% RGB error
100
75
50
25
0
Comparison
Total UMIs (millions)
9.8
13.0
original
Syrah
Mean % error
26.4
2.5
original
Syrah
Syrah output
Syrah output
Syrah % error
