# Supplementary material for: Syrah: a pipeline to maximize spatial transcriptomics data output
Source: G3 (Bethesda). 2026 May 4;16(7):jkag107. doi: 10.1093/g3journal/jkag107 (PMC13334189; doi:10.1093/g3journal/jkag107)
Supplement: jkag107_Supplementary_Data [file jkag107_supplementary_data.zip › Supplementary_Figure_Legends_G3-2025-406488R1.docx]

## **Figure S1 caption: Read loss during analysis of Drop-seq single-cell versus Slide-seqV2 data.** Comparison of percent of reads remaining at different analysis steps for Drop-seq versus Slide-seqV2 planarian data aligned to the SMED20140616 reference (Tu et al. 2015; Fincher et al. 2018).

**Figure S1 alt test:** Barplots depicting the percent of reads retained at several stages during Drop-seq or Slide-seqV2 data processing, showing a dramatically larger loss during the barcode matching step for Slide-seqV2 data.

**Figure S2 caption:** **Examples of reads rescued by Syrah**. a) These examples depict twelve reads from our planarian dataset and show how each was handled for the original versus Syrah-processed version. Orange bars above reads indicate sequences extracted by original pipeline; orange arrows point to the fates of those reads in the original pipeline. Blue bars below reads indicate sequences extracted by Syrah, arrows point to the fates of those reads. The ID of each read from the sequencing run is shown in the final column. b) Slide embeddings for original (left) and Syrah-processed (right) data, with beads under tissue sections in darker gray. Highlighted beads correspond to reads in part a).

**Figure S2 alt text:** Diagrams showing the results of barcode matching both with and without Syrah processing for 12 reads from the planarian dataset, and comparing their computed locations on the Slide-seq puck. Subfigures are labeled a through c.

**Figure S3 caption:** a) Percent change in total beads (top), UMIs (middle), and genes (bottom) for original versus Syrah-processed data across a variety of Slide-seqV2 and Curio Seeker datasets with various minimum UMIs/bead cutoffs (top x-axis). Bars indicate 95% confidence interval of Poisson rate test; asterisks denote p-value < 0.05. b) Boxplot of PHRED quality scores for mouse reads categorized by location in final data. Horizontal bar indicates median, boxes stretch from first to third quartile, and whiskers indicate smallest/largest non-outlier values. Outliers are included in calculations but omitted from plot for visual clarity. c) Plot of standard deviation of principal components for original versus Syrah-processed mouse data. d) UMAPs of mouse Syrah-processed data colored by clusters generated using default parameters. Left UMAP shows all beads, right UMAP shows beads specific to Syrah-processed data. e) Boxplot of PHRED quality scores for chick reads categorized by location in final data. Horizontal bar indicates median, boxes stretch from first to third quartile, and whiskers indicate smallest/largest non-outlier values. Outliers are included in calculations but omitted from plot for visual clarity. f) Plot of standard deviation of principal components for original versus Syrah-processed chick data. g) UMAPs of chick Syrah-processed data colored by clusters generated using default parameters. Left UMAP shows all beads, right UMAP shows beads specific to Syrah-processed data. h) Boxplot of PHRED quality scores for human reads categorized by location in final data. Horizontal bar indicates median, boxes stretch from first to third quartile, and whiskers indicate smallest/largest non-outlier values. Outliers are included in calculations but omitted from plot for visual clarity. i) Plot of standard deviation of principal components for original versus Syrah-processed human data. j) UMAPs of human Syrah-processed data colored by clusters generated using default parameters. Left UMAP shows all beads, right UMAP shows beads specific to Syrah-processed data.

**Figure S3 alt text:** A variety of plots showing the result of using Syrah on a datasets from a variety of organisms, displaying increases in beads, UMIs, genes, and overall data quality, including downstream analyses for mouse, chick, and human datasets. Subfigures are labeled from a to j.

**Figure S4 caption:** a) Runtime for Syrah’s barcode correction step with various numbers of reads. At low read counts read/write processes dominate and run time is roughly constant. With higher read counts run time scales linearly with the number of reads. b) Average reads processed per minute by Syrah’s correct_barcodes() function across a variety of platforms. Points are individual data, diamonds represent mean values, and error bars show the mean standard error.

**Figure S4 alt text:** Dotplots labeled a and b showing the run time for Syrah’s barcode processing step increasing linearly with millions to hundreds of millions of reads and the number of reads processed per minute in a variety of computational environments.

**Figure S5 caption:** a) Bar plots showing the percent increase in beads (top), UMIs (middle), and genes (bottom) provided by additional sequencing, Syrah processing, or the combination of additional sequencing and Syrah processing across a variety of minimum UMIs/bead cutoffs (top x-axis). Error bars indicate 95% confidence interval of Poisson rate test versus non-Syrah corrected data from a single sequencing run; asterisks denote p-value < 0.05. b) Raw counts of beads (top), UMIs (middle), and genes (bottom) for original or Syrah-processed singly- or doubly-sequenced data across a variety of minimum UMI cutoffs (top x-axis). The color legend applies to both plots.

**Figure S5 alt text:** Barplots comparing singly- and doubly-sequenced data with or without Syrah processing. Subfigures labeled a and b.

**Figure S6 caption:**  a) Bar plots of total beads (top), UMIs (middle), and genes (bottom) for original and Syrah-processed versions of data across various deletion rates (bottom x-axis) and minimum UMIs/bead cutoffs (top x-axis). Asterisks denote Poisson rate test for original versus Syrah-processed data with p-value < 0.05. b) Plots of standard deviation of the first 25 principal components for standard versus Syrah-processed data for a variety of deletion rates.

**Figure S6 alt text:**  Plots showing the results of the in silico experiment using artificially introduced deletions across a variety of deletion rates and UMI cutoffs. Subfigures are labeled a and b.

**Figure S7 caption: Syrah improves quantity and fidelity of synthetic image-based data.** a,b) Two image-based de novo spatial datasets. Top left is starting image data. Bottom left shows total UMIs and mean percent RGB color error for both original and Syrah-processed versions. Center-left column shows slide embedding for original (top) and Syrah-processed (bottom) data colored by individual bead RGB values. Center-right column is detail inset of slide embedding. Right column shows slide embedding of original (top) and Syrah-processed data colored by % RGB error for each bead.

**Figure S7 alt text:** Plots and images showing the results of Syrah processing on image-based datasets generated from images of a bowl of fruit and the painting “Mona Lisa.” Subfigures are labeled a and b.
