# Supplementary figures and images for: Syrah: a pipeline to maximize spatial transcriptomics data output
Source: G3 (Bethesda). 2026 May 4;16(7):jkag107. doi: 10.1093/g3journal/jkag107 (PMC13334189; doi:10.1093/g3journal/jkag107)

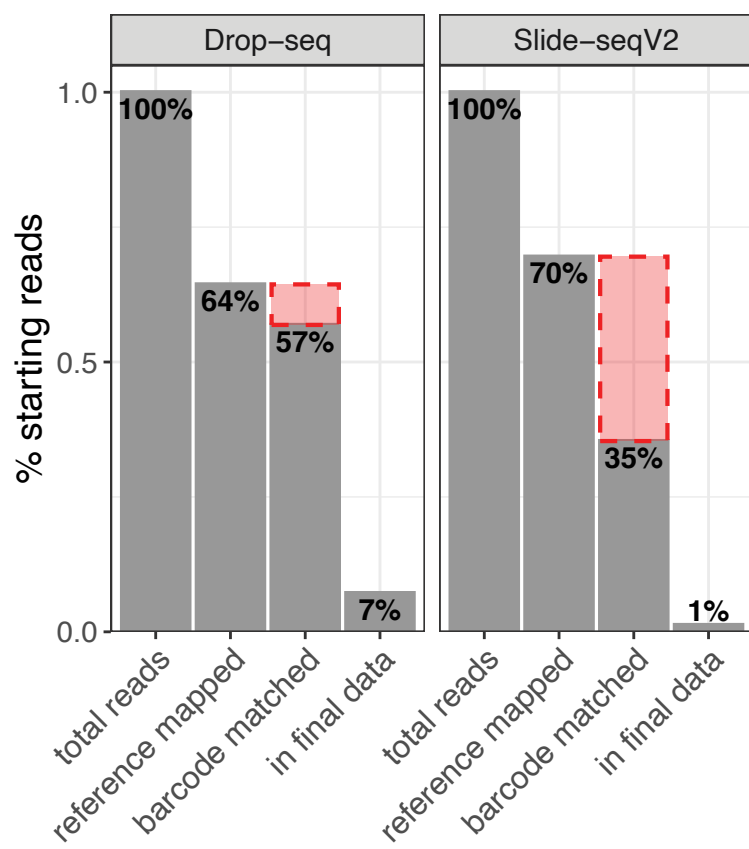

Supplement: jkag107_Supplementary_Data [file jkag107_supplementary_data.zip › Figure_S1_G3-2025-406488R1.pdf]

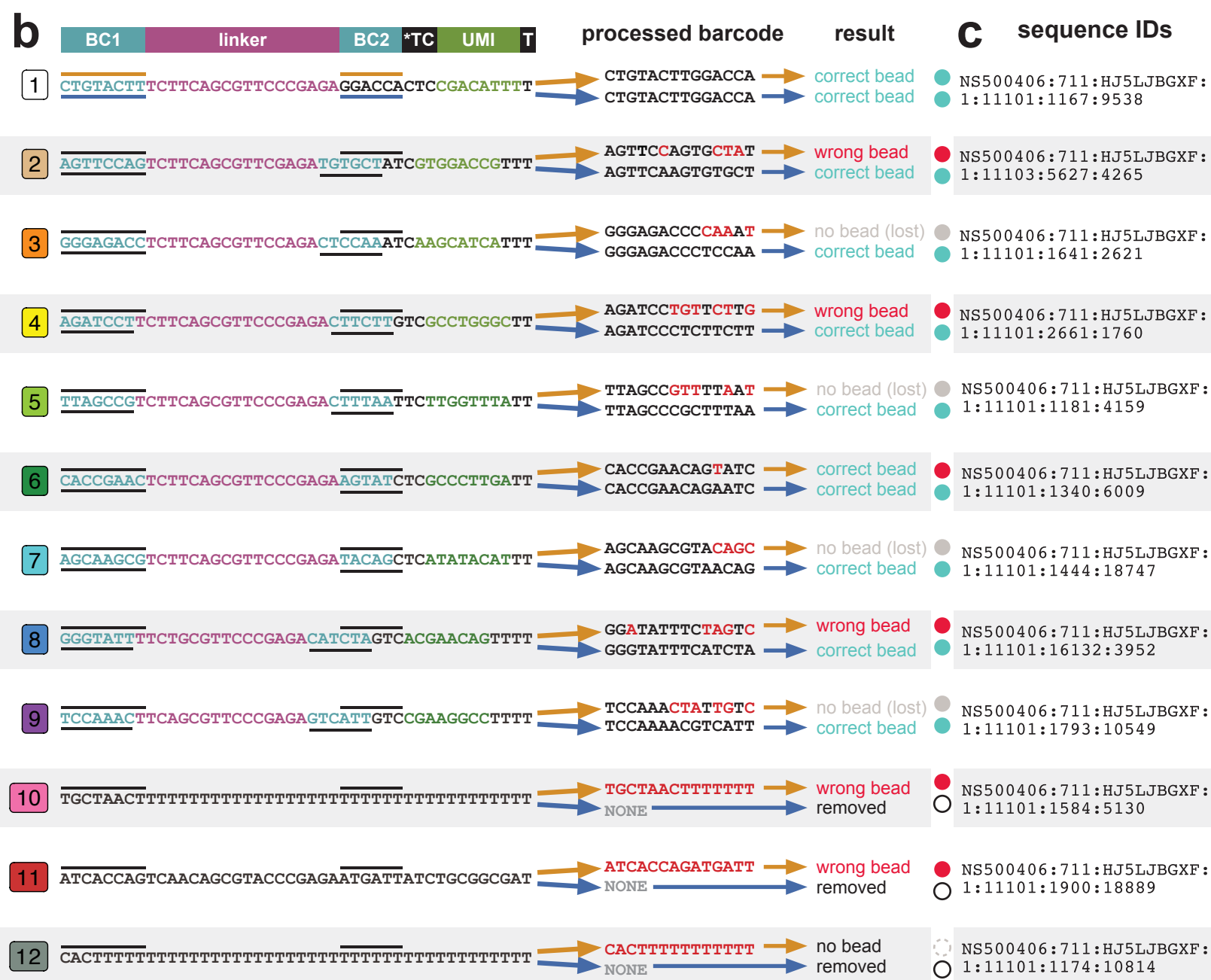

Supplement: jkag107_Supplementary_Data [file jkag107_supplementary_data.zip › Figure_S2_G3-2025-406488R1.pdf]

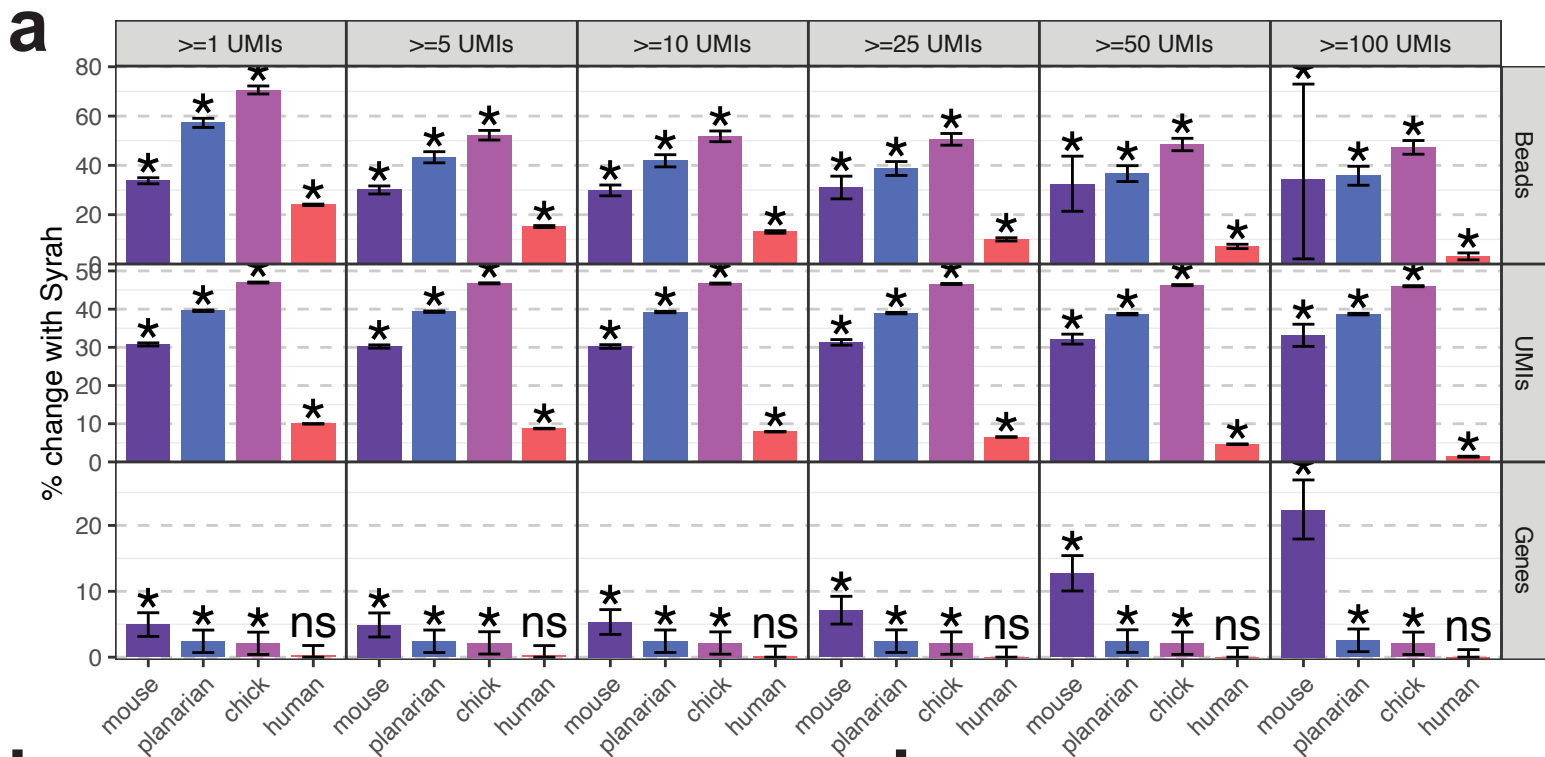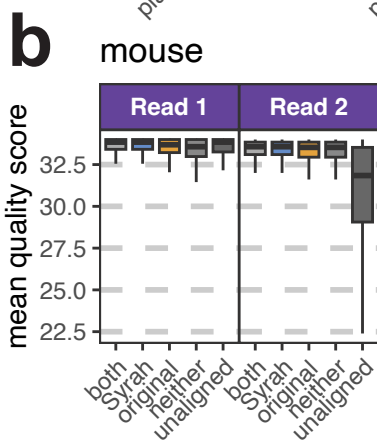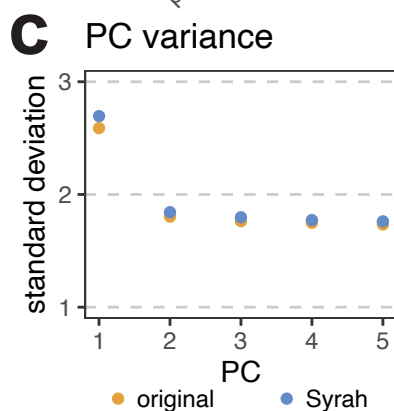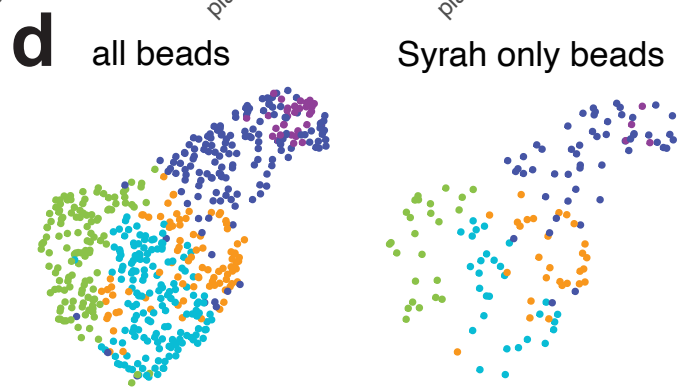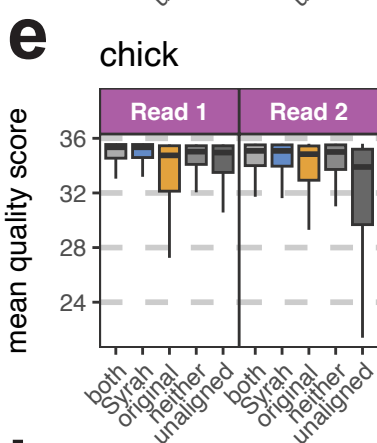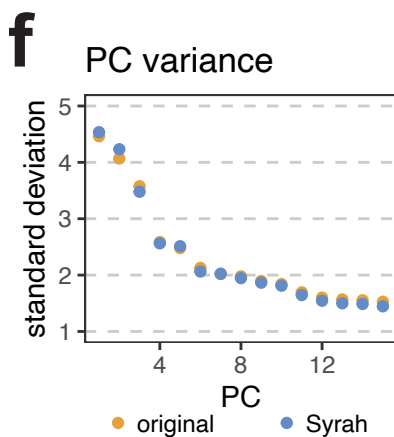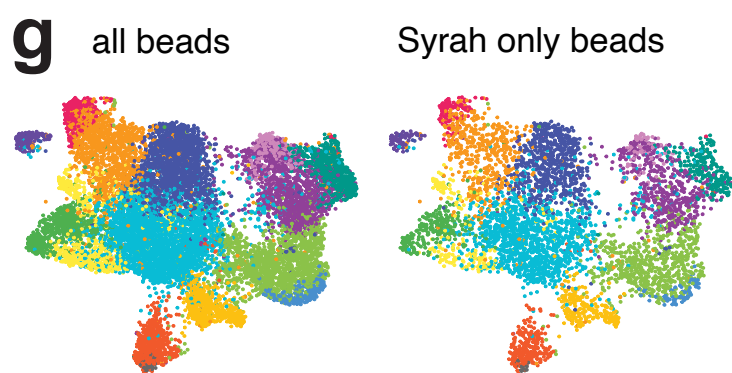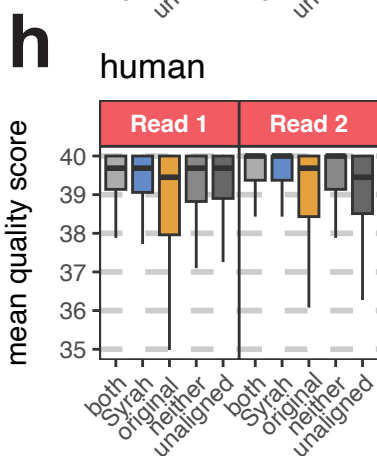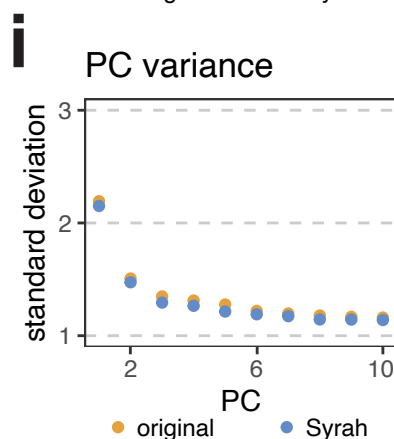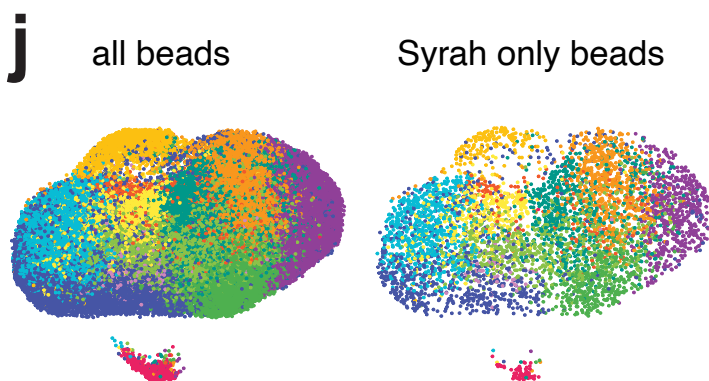

Supplement: jkag107_Supplementary_Data [file jkag107_supplementary_data.zip › Figure_S3_G3-2025-406488R1.pdf]

**a**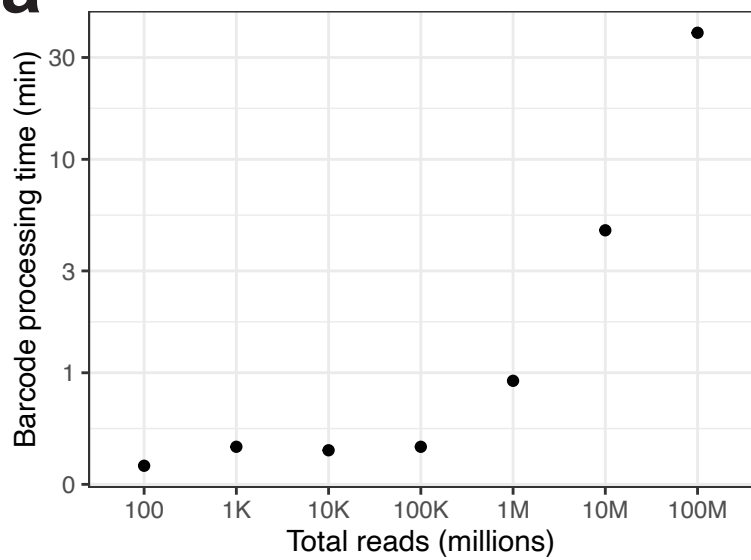**b**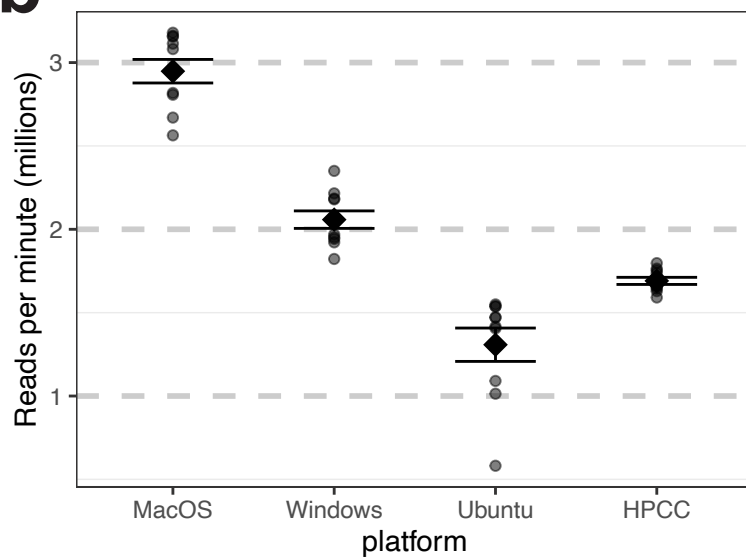

Supplement: jkag107_Supplementary_Data [file jkag107_supplementary_data.zip › Figure_S4_G3-2025-406488R1.pdf]

**a**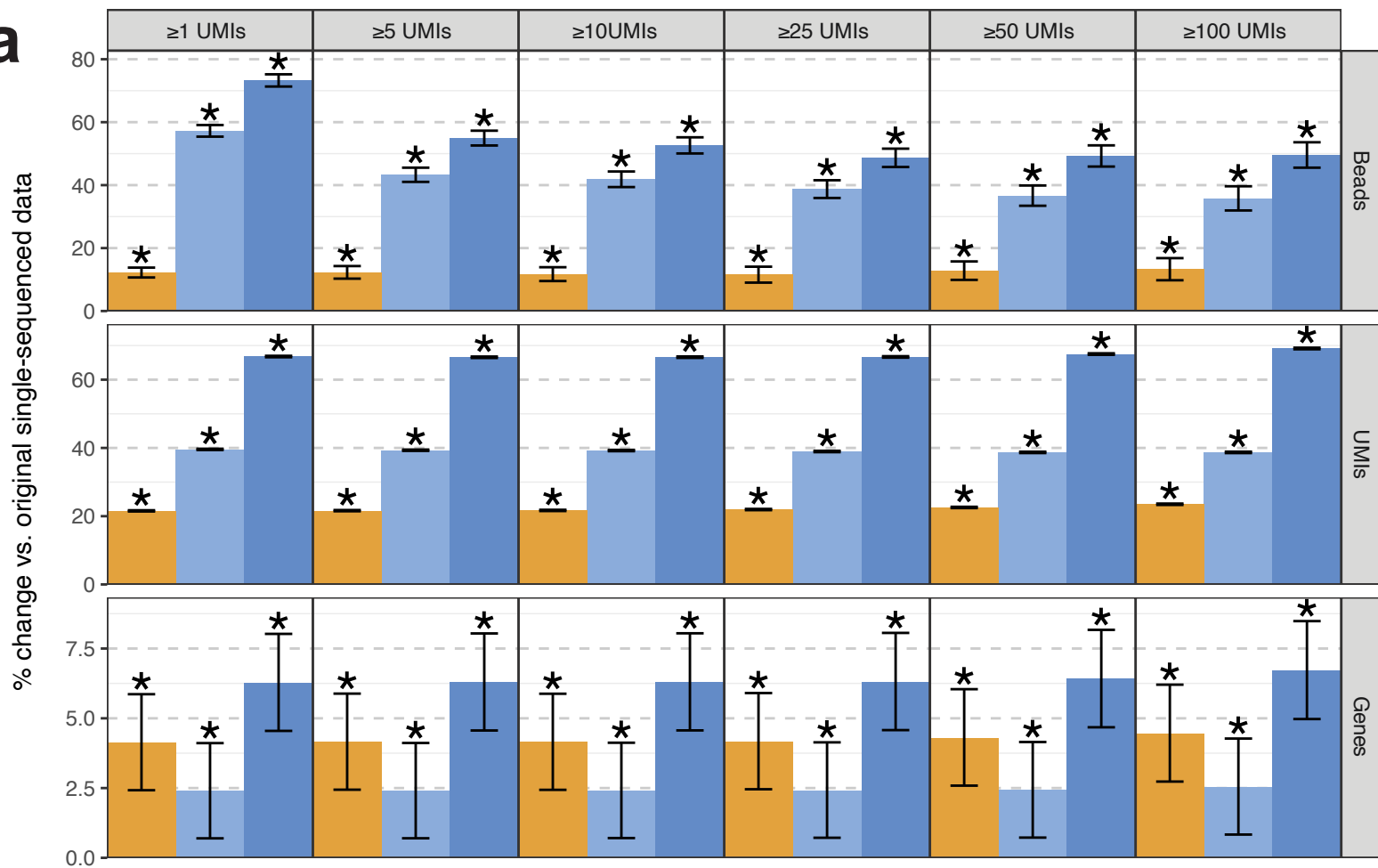**b**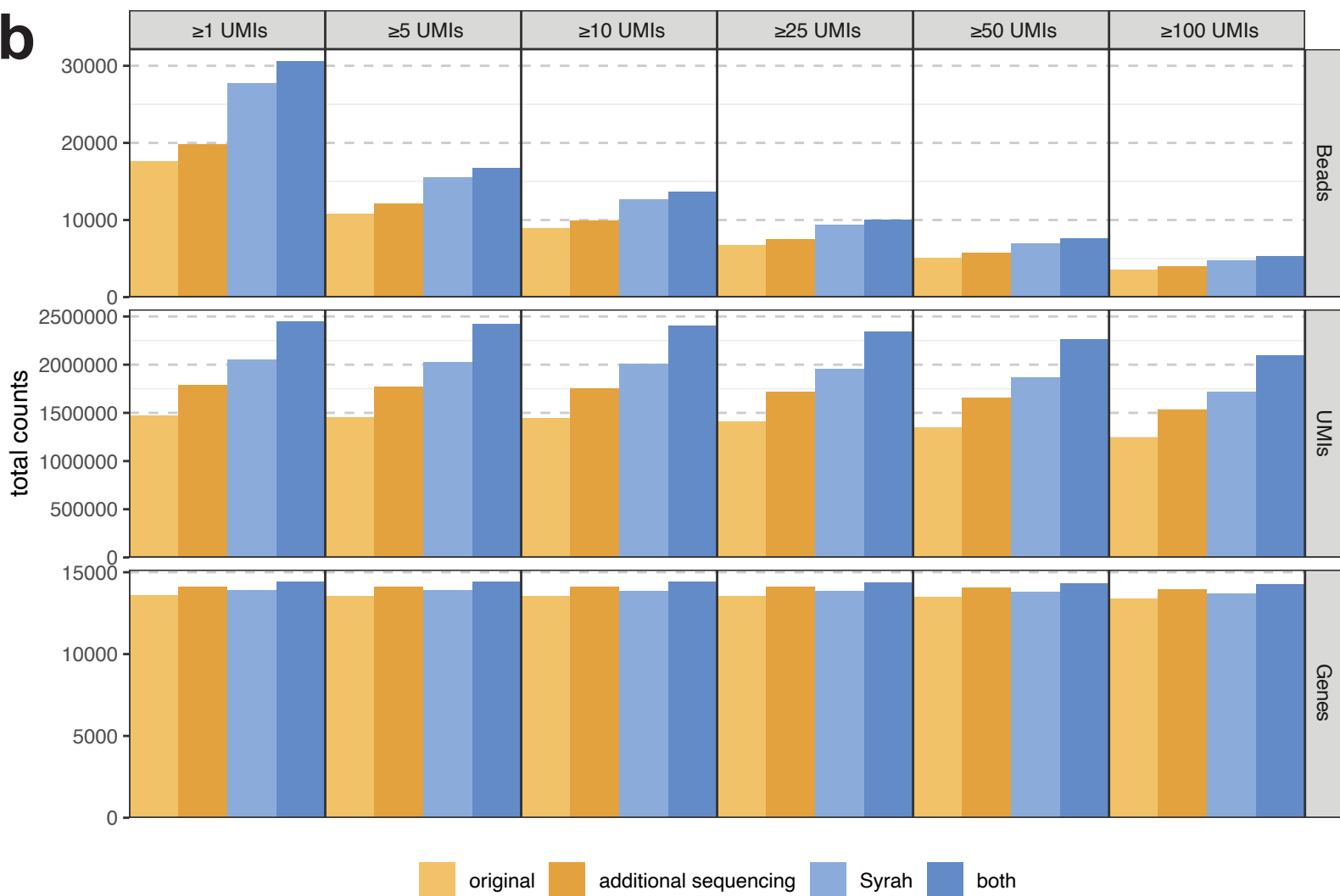

Supplement: jkag107_Supplementary_Data [file jkag107_supplementary_data.zip › Figure_S5_G3-2025-406488R1.pdf]

**a**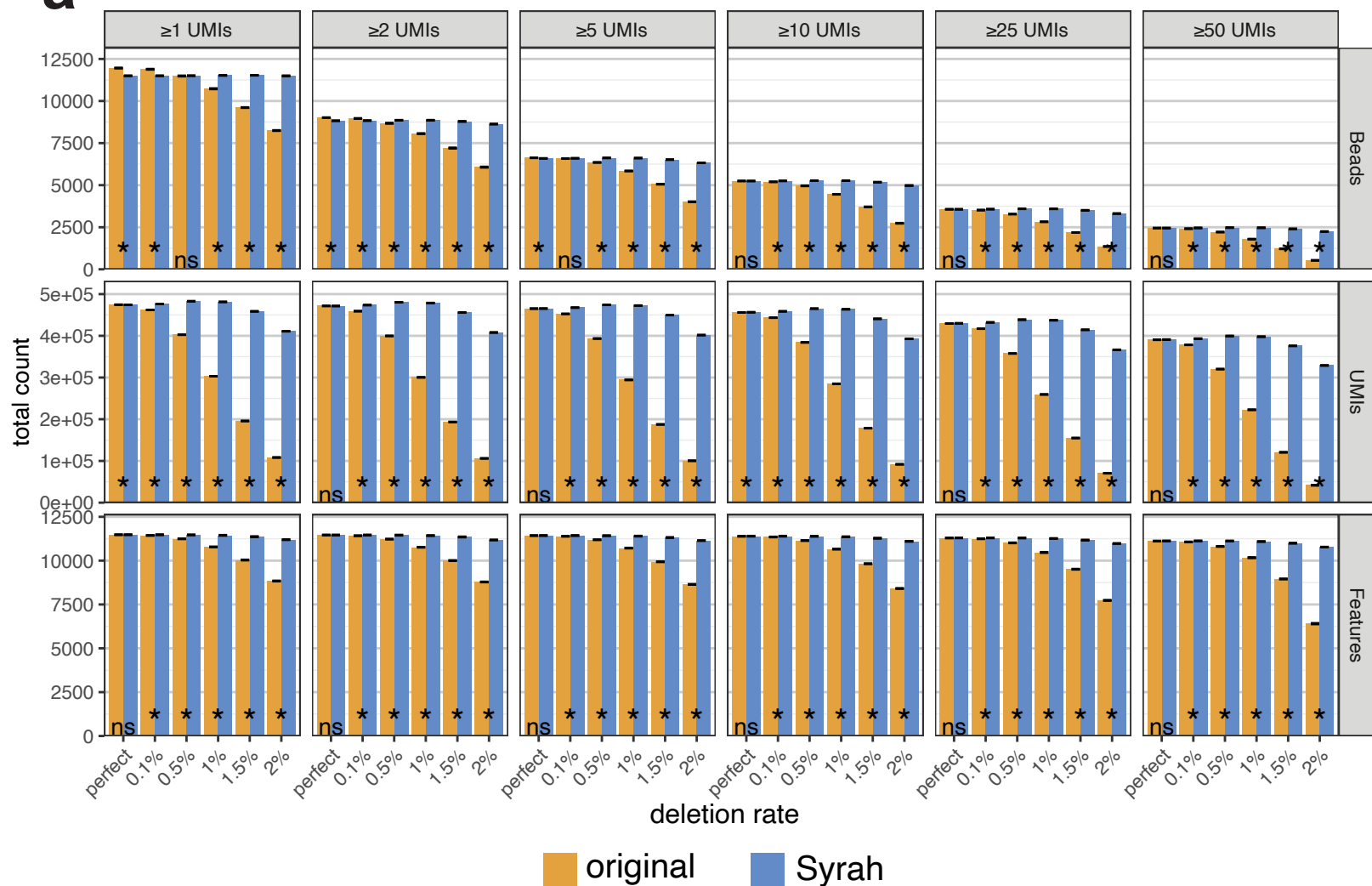**b**

PC variance by deletion rate

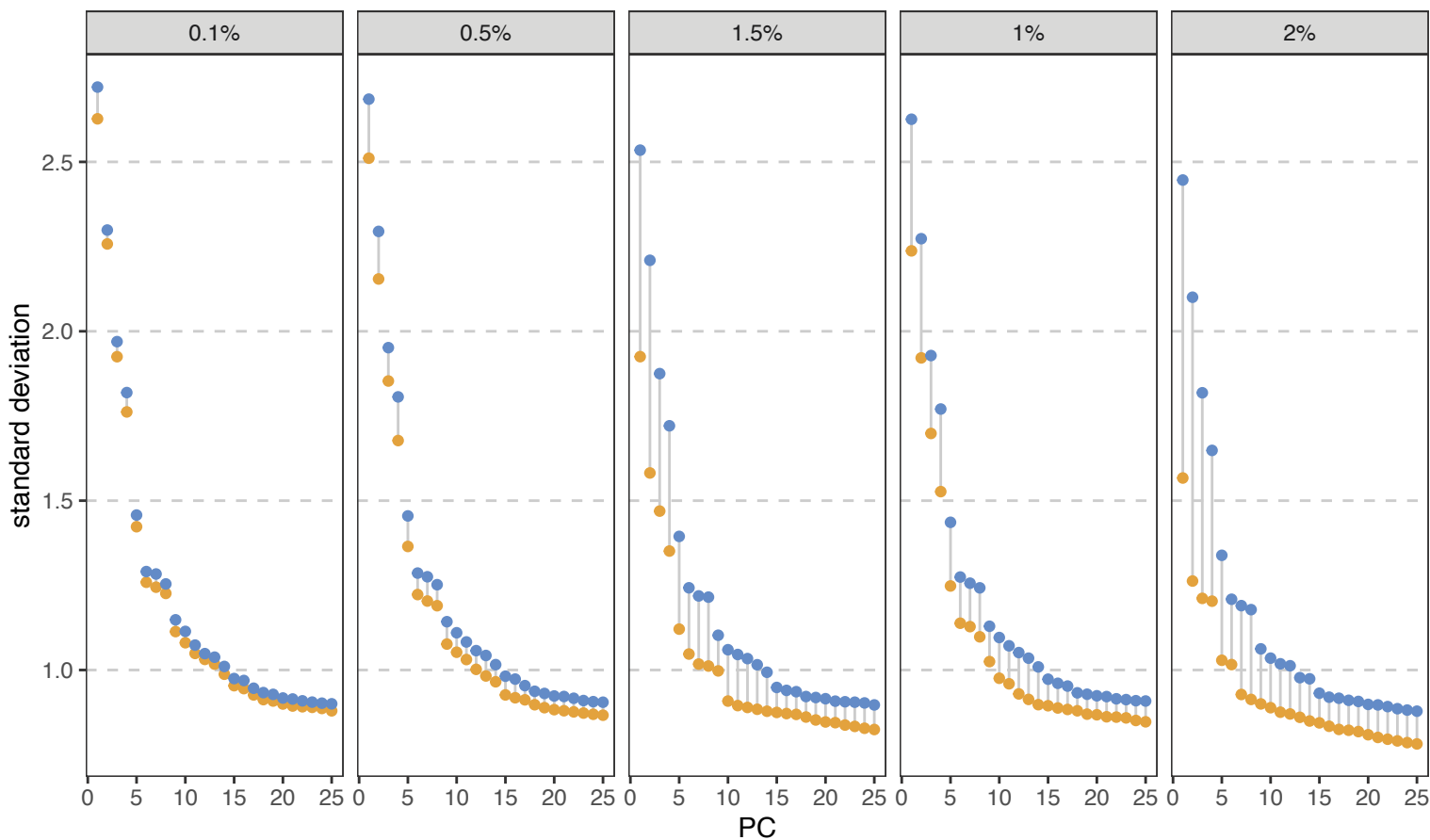

Supplement: jkag107_Supplementary_Data [file jkag107_supplementary_data.zip › Figure_S6_G3-2025-406488R1.pdf]
